# Kidney Care during COVID-19 in the UK: Perspectives of Healthcare Professionals on Impacts on Care Quality and Staff Well-Being

**DOI:** 10.3390/ijerph19010188

**Published:** 2021-12-24

**Authors:** Archontissa Maria Kanavaki, Courtney Jane Lightfoot, Jared Palmer, Thomas James Wilkinson, Alice Caroline Smith, Ceri Rhiannon Jones

**Affiliations:** 1 Leicester Kidney Lifestyle Team, Department of Health Sciences, University Hospitals of Leicester NHS Trust, University of Leicester, Leicester LE17RH, UK; mkanavak@affil.duth.gr (A.M.K.); courtney.lightfoot@leicester.ac.uk (C.J.L.); Jared.Palmer@nottingham.ac.uk (J.P.); t.j.wilkinson@leicester.ac.uk (T.J.W.); alice.smith@leicester.ac.uk (A.C.S.); 2 Department of Neuroscience, Psychology and Behaviour, University of Leicester, Leicester LE17RH, UK

**Keywords:** COVID-19, telemedicine, healthcare professionals, care quality, kidney service

## Abstract

In light of the rapid changes in healthcare delivery due to COVID-19, this study explored kidney healthcare professionals’ (HCPs) perspectives on the impact of these changes on care quality and staff well-being. Fifty-nine HCPs from eight NHS Trusts across England completed an online survey and eight took part in complementary semi-structured interviews between August 2020 and January 2021. Free-text survey responses and interviews were analysed using inductive thematic analysis. Themes described the rapid adaptations, concerns about care quality, benefits from innovations, high work pressure, anxiety and mental exhaustion in staff and the team as a well-being resource. Long-term retention and integration of changes and innovations can improve healthcare access and efficiency, but specification of conditions for its use is warranted. The impact of prolonged stress on renal HCPs also needs to be accounted for in quality planning. Results are further interpreted into a theoretical socio-technical framework.

## 1. Introduction

Since March 2020, the COVID-19 pandemic has resulted in a prolonged overload of the National Health System (NHS) in the UK, which has had to treat patients at excessive numbers during the peaks of the pandemic, whilst reconfiguring existing service provision to ensure patient and staff safety. Rapid adaptations took place in healthcare delivery, including staff redeployment to manage the influx of patients, and services done remotely or ceased for infection control and prevention [1,2,3].

Healthcare Professionals (HCPs) have faced numerous occupational stressors during the pandemic including increased workloads, changes in roles, and concerns about patient care. These have been exacerbated by a lack of knowledge and information, rapidly changing procedures, and concerns about lack of personal protective equipment, staff shortages, and risk of infection for themselves and their families [1,4,5,6,7]. Consistent with evidence on the negative psychological impact of previous epidemics on HCPs [8], there have been reports of mental distress and mental exhaustion among staff [1,9] with a high prevalence of anxiety, depression and insomnia among HCPs during COVID-19 [10].

One study with kidney HCPs during and soon after the first global lockdown, highlighted that staff experienced multifaceted work-related stress [11] and although many showed resilience and felt supported by their department, they were at risk of emotional exhaustion [12]. A key protective factor minimizing the impact of work-related stress in HCPs is perceived organizational support [13]. Healthcare organizations that have supportive structures in place where HCPs feel cared for can foster individual wellbeing and resilience [14]. In turn, HCPs play a vital role in organizational resilience—an organization’s capacity to adapt, recover, and transform from sudden disruptions [15]. Resilient organizations have good systems and processes in place to monitor care quality and safety as well as supporting staff. HCPs’ poor mental health and exhaustion has been linked to poorer outcomes in quality of care for patients [16,17] and patient satisfaction particularly when staff perceive they have not been supported by their organization [18].

Studies researching HCPs experiences in kidney services are limited, particularly qualitative studies capturing the in-depth complexities of real-time experiences during the different phases of the pandemic [11]. However, the impact of COVID-19 on staff well-being has been well recognised by national organizations (e.g., UKKA, 2021) in the UK, and has resulted in the development of resources promoting mental well-being. The demands on HCPs in kidney services are complex. Kidney care involves regular contact with clinically vulnerable individuals often with multi-morbidities. HCPs provide long-term care through regular hospital appointments for renal replacement therapy (RRT) and monitoring of disease progression, which makes it a significant challenge for kidney care services to balance care quality with patient safety [19,20]. In addition, COVID-19 reportedly impacts on kidney function in the general population, with acute kidney injury and the need for RRT a further complication, while chronic kidney disease patients are at higher risk of COVID-19 infection and adverse outcomes [20,21,22,23]. The COVID-19 pandemic and associated lockdowns has disrupted kidney services and the redeployment of HCPs meant disruptions to RRT, limited access to kidney transplants and other surgical procedures. The rapid innovation of services to tele-nephrology has moved much of the routine medical care away from hospital settings into patients’ homes [24]. Incorporating telemedicine in kidney care has been a priority prior to the COVID-19 pandemic but the efficacy and safety of this has yet to be determined particularly for those patients with advanced kidney disease [25,26,27]. Despite this, there appears to be good acceptability of telemedicine in kidney care for patients and their significant others, an important component in the adoption and delivery of service innovations [28,29].

The present study was initiated by a kidney research group in response to a COVID-19 outbreak, to map and explore the impacts of a new developing pandemic on kidney care, utilizing an ongoing multicenter mixed-methods study. This study’s specific aim was to understand the impacts of (a) the pandemic on the psychological wellbeing of HCPs in kidney services and (b) the impacts on care delivery during the pandemic from HCPs perspectives. Data were collected following the first lockdown (eased restrictions) until after the end of the second lockdown in the UK.

## 2. Methods

**Study design.** This is the DIME-CV sub-study of the multicentre DIMENSION-KD observational study (ISRCTN84422148). DIME-CV is a mixed-methods study involving kidney patients, their significant others and HCPs. The present paper reports the qualitative data, i.e., free-text survey responses and telephone interviews, from HCPs and is part of a series of reports on this study [28,29]. The qualitative research design has an interpretative underpinning epistemology, and the Consolidated Criteria for Reporting Qualitative Research statement informs study reporting (Supplementary Material S1).

**Ethics.** The study was approved by the Health Research Authority and Leicester Research Ethics Committee (Ref. 18/EM/0117), UK.

**Participant selection, settings and procedures**. HCPs involved in kidney care from eight NHS Trusts across the UK were invited to take part in the survey by local clinical research facilitators and managers. There were no exclusion criteria (number, role, etc.). Participants filling in the survey could opt in for an interview and all those who did were later invited to attend one. Survey data collection (Jisc Online Surveys, Bristol, UK) took place between August and December 2020. One to one telephone interviews took place between December 2020 and January 2021. Interviews were conducted by AMK, female, white (non-British), psychologist, with training and post graduate experience in qualitative methods. The researcher had no specific or strong prior assumptions about the research topic. Participants were sent written information about the survey and the survey link by the local collaborators and signed an online consent form before filling in the survey. Information about the interview study was emailed by the researcher conducting the interviews and introduction of the researcher (credentials, role) was given verbally during the telephone appointment. Prior to confirming a telephone appointment, participants signed a separate online consent form. Interviews were recorded using a digital voice recorder and field notes were kept following each interview.

The bespoke free text survey questions reported in the present paper explored the perceived (1) impacts of COVID-19-related changes on care delivery, (2) their benefits and (3) drawbacks; impacts on oneself and the team with regard to (4) well-being (psychological), (5) behaviours, and (6) communications. Given the COVID-19 restrictions and the high workload of NHS staff, online free-text questions were deemed appropriate as they are easily accessible, offer flexibility regarding time-commitment, but can also facilitate rich disclosure of experiences (Braun et al., 2020). Interviews aimed to further explore experiences and views on the impact of COVID-19 on kidney care, challenges and needs for HCP support and ways to move forward in kidney care (Interview Schedule Supplementary Material S2).

**Data analysis.** Inductive thematic analysis [30,31] was applied, first to survey responses, next and separately to interview transcripts in an iterative process (AMK). Survey responses (Excel) were imported in NVivo 14 (QSR International©) and after familiarization with data, codes were assigned to all text. Unit of analysis was whole or part of a free-text response with a complete meaning. Codes were a close description of the content. Next, codes were compared with one another, and similar codes were merged or grouped together in first and second order subthemes, then themes and findings were written up. Debriefings with a co-investigator (CRJ), an occupational psychologist with experience of staff wellbeing and organizational psychology, who studied the subthemes and their content. Alternative themes and organizing framework were examined. At this point a care quality focus was adopted, informed by the organizational resilience framework. Next, an auditor (CJL), sport and exercise scientist with experience in research in kidney healthcare, reviewed the content of analysis. Following this feedback, findings were further refined, i.e., themes were reduced and subthemes merged so that the findings become more focused. Interview transcripts were analysed at this point in NVivo as described above and were embedded in the reported findings.

**Trustworthiness**. The sample of participants was appropriate for the study, i.e., HCPs had direct experience of the topic under study. Analysis was grounded in the data and an audit trail links back to the data as evidenced by the original quotes provided. Other processes to ensure trustworthiness of the findings were peer debriefing, external audit, thick description, negative case analysis, reflexivity.

## 3. Results

Fifty-nine eligible HCPs took part in the survey out of the 1167 invited. Of the 40 who opted in for an interview, eight attended (participant characteristics in Table 1). Number of participants responding to free-text questions was variant, with 57–58 responding to inquiries about impacts on communication/behaviour/psychological, and 39–40 to inquiries about changes in care delivery, benefits and drawbacks. Interview time ranged from 22 to 67 min (mean = 37 min). Four themes were consistent across participant responses. Themes, subthemes and original quotes from survey (S) and interviews are presented below (more supporting quotes in Supplement S3).

### 3.1. Rapid Changes and Adaptation in Care Delivery

The reported changes varied among participants. Rapid change and innovations took place in kidney care following the initial shock of the emerging crisis. The few accounts of how changes were initiated described both top-down and individual/team initiatives.

#### 3.1.1. Reduced Patient Contact and Services

Most participants referred to reduced face-to-face patient contact across nephrology services, i.e., remote outpatient appointments, mostly via telephone, and cessation of or socially distanced inpatient visits. Face-to-face appointments were still conducted, when clinically indicated and absolutely essential. Fluctuation in number of face-to-face appointments depending on national restrictions was also reported. Other changes in services included minimal haemodialysis care, suspension of patient information events and groups, some patient education and interventions done by telephone and mail, suspension of home visits and other support services in the community, changes in duties and staff mobility between clinics and wards, including COVID-19 wards, to meet demands and staff shortage.

*During inpatient ward rounds less likely to sit at the bedside and talk Less physical patient contact unless absolutely necessary less face-to-face appointments in clinic.*

(doctor (S), 51y)

#### 3.1.2. Remote Team Communication

Some participants reported staff and multidisciplinary team (MDT) communication changed to remote, i.e., virtual, via emails and telephone.

*Face-to-face meeting with members of study team has beenstopped. We communicate using zoom, emails and phone,* etc. *We are still able to have proper communication with the help of different technology.*

(nurse (S), 46y)

#### 3.1.3. Infection Control and Prevention

This was often noted as a pertinent change, including use of personal protective equipment, increased self-awareness of potential symptoms, being more cautious and constant staff updates on guidelines.

*And even now, we have a thermometer and a sats machine, a portable one, so every time we go inside, we need to make sure that everything is safe. So yeah, that changed.*

(Interview 1, specialist nurse)

### 3.2. Impacts on Care Quality

This theme includes the direct and/or indirect issues raised and assessments made about COVID-19-related changes in renal care.

#### 3.2.1. Drawbacks and Concerns for Care Quality

The quality of care delivered to patients was routinely highlighted as a cause for concern and one of the most prevalent topics in participants’ responses.

##### Difficulties in Patient Assessment and Communication

The challenges of remote patient assessment and monitoring was a pressing concern for most participants, who found it unsatisfactory. The absence of a physical examination and visual cues, non-verbal communication and objective clinical measurements and a reliance on patients self-reporting their condition meant missing important information. This issue was even more intensified for patients with advanced or deteriorating kidney disease, and patients in haemodialysis due to the condition’s complexities, and this could impact on appropriateness and timeliness of treatment and diminish the level care provided.

*However, for some patients, telephone clinics mean problems are not being sorted, not getting monitoring to know if intervention is required.*

(doctor (S), 31y)

*They [dialysis patients] tend to be complex, some have hearing difficulties, and others are non-English speakers. I feel that I cannot give them the level of care that I would be able to if I were visiting the unit/seeing them face to face.*

(doctor (S), 38y)

Depending on their role, interview participants identified different patient groups as more impacted, e.g., dialysis patients, younger inpatients, older outpatients. Due to a fear of infection of COVID-19 some patients had reportedly concealed their condition to avoid having to attend hospital appointments leading to deterioration and acute episodes. Clinics and service reductions or cancellations, staff shortages and lack of face-to-face MDT communication also created delays in patients receiving appropriate treatment leading to sub-optimal care.

*Patients not admitting to symptoms on the phone and becoming unwell leading to acute episodes, visits to renal assessment unit and admission. Patients starting renal replacement therapy sooner than expected as they have not been monitored as effectively.*

(nurse (S), 59y)

Other issues included care being more fragmented and less personalised, limited space capacity for face-to-face appointments, so less family involvement. Certain issues were relevant to local procedures and not consistently reported across sites, like difficulties in getting blood results on time.

More than one in three participants described difficulties in HCP-patient communication. This was particularly challenging with certain patient groups such as those with hearing problems, typically elderly, and non-English speakers, which may have led to inequalities in care provision. Other communication challenges were related to building a rapport, introducing treatment options to new patients, dialysis education, breaking bad news, communication via other HCPs, technical difficulties, IT-related disruptions in communication and confidentiality issues.

*Building rapport is key to our assessment being patient friendly. The physical barrier of the mask and visors do not help us reassure our patients of confidentiality as they divulge very personal information on a ward.*

(occupational therapist (S), 51y)

##### Increased Work Pressure

Most participants reported increased workload and work pressure. Reasons given included changes in procedures, covering for staff absences as staff contracted COVID-19 or were shielding, increased demand for inpatient care including COVID-19 wards, increase in administrative tasks, difficulties in team remote communication and coordination.

*Everyone is ‘busier’—it is certainly a perception and it has factual basis e.g., staffing challenges due to sickness/isolation. Serious nursing shortage; pressured demand for ‘bank’ working.*

(doctor (S), 73y)

#### 3.2.2. Efficiencies and Benefits of Implemented Changes

Participants did note the positive impacts of these changes in healthcare working practices, although these were cited less frequency than the drawbacks and challenges.

##### Efficiencies and Benefits for Clinical Practice

The benefits of remote and telemedicine appointments, particularly the convenience for patients, was frequently discussed. However, for this benefit to be maximised the patient’s condition needed to be well-managed, with stable blood

*Managing patients by blood tests and telephone calls has been easier for some of them. Perhaps highlighted we can see some patients less frequently so long as they have regular blood tests. Less need for patient transport.*

(nurse (S), 59y)

Time saving was another cited benefit, using telephone as a “triage” for arranging face-to-face appointments; more flexible working arrangements replaced “presentism in NHS” and allowed doctors to meet multiple commitments along with telephone consultations and for some less time altogether spent on consultations. Other efficiencies included improved collaboration between primary and secondary care and rapid innovations, such a highly regarded drive-through phlebotomy service.

*I mean what the pandemic has shown is that we can work with primary care very quickly and very effectively.*

(Interview 8, consultant)

##### Crisis Enabling Innovation

Some participants highlighted how under the COVID-19 crisis mode new models of care were rapidly introduced and tested in a complex healthcare system. COVID-19 “forced” HCPs to think differently about their roles and both patients and HCPs “to accept change”. Innovations were implemented and initiatives developed, some of which had been previously stuck in resistant-to-change mechanisms, and evidence for/against new models of care became readily available. This aspect of COVID-19 impact was explicit in interview data, less so on survey responses.

*And the guy that [created the new service], he’s been wishing to do it for eight years, he’s been saying this’ll be amazing for patients…*

(Interview 5, specialty doctor)

##### COVID-19 Safety

Limiting COVID-19 transmission and ensuring patient and staff safety in care delivery was discussed by some HCPs. They acknowledged that the infection prevention measures taken were necessary and would need to continue in the future leading to a “new normal” rather than a “back to normal”.

*The changes have benefited both staff and patients in the fact of reducing the risk factor of the virus.*

(nurse (S), 55y)

### 3.3. Impacts on Staff Well-Being

The negative psychological impacts of working during the pandemic were cited often across all HCP groups.

#### 3.3.1. Increased Stress and Anxiety

Most participants referred to increased levels of generalised anxiety and stress experienced amongst staff.

*Higher levels of general stress amongst the clinical team.*

(doctor (S), 39y)

Whilst a number of participants did not expand on the sources of their anxiety, others referred to a fear of becoming infected with COVID-19 at work and then transmitting it to patients and family, which was particularly heightened at the peaks of the pandemic and in clinically vulnerable staff. Other sources of anxiety included poor communication, negative workplace behaviours and heightened emotions. Work pressures that caused anxiety included: covering for staff absences, adjusting to rapid changes in procedures, roles and services and the loss of MDT support, especially in the wards. Concern for patients and the distress that caused was a source of anxiety particularly for those deployed treating COVID-infected patients and the deaths of both patients and staff members. Concerns about patient support provisions not being adequate was also a source of anxiety and the frustration of not seeing patients face to face. This was underpinned by the general climate of uncertainty in addition to disruption in well-being resources outside work.

*There is anxiety relating to uncertainty and a demoralization as so many planned activities are cancelled and contact with friends and family is reduced.*

(doctor (S), 60y)

#### 3.3.2. Mental Exhaustion, Negative Affect and Fatigue

A sense of weariness, fatigue and mental exhaustion from the prolonged crisis and uncertainty was expressed by many participants. They described how staff resilience had diminished over time and the emotional impact of COVID-19 reporting feeling “tired”, “drained”, “fed up”, “overwhelmed” at times, “disillusioned”, “unhappy”, “angry”, having sleep problems. Individual and collective struggles were noted including “ward burnout”.

*I feel exhausted after shifts and have worked late on regular basis to try and keep up. I have booked more random days off work just to recuperate. I take pain relief every day without fail. [] General unhappiness is the new normal sadly.*

(occupational therapist (S), 51y)

### 3.4. Team and Organizational Support

For many participants, peer and team support was important in managing work stress. Organizational support was identified by fewer participants.

#### 3.4.1. Team Support and Teamwork

Many participants highlighted the teamwork, peer and team support they had experienced, which had remained constant or improved during the pandemic.

*Team morale and teamwork improved—felt there was a sense of ‘this is a crisis so we have to get on with it’.*

(ward manager (S), 46y)

Such accounts included team coming together, stepping up, being flexible, and showing increased tolerance, greater compassion and appreciation of colleagues. Closer staff relationships were a positive and provided a buffering effect during this “horrible period”. Emphasis on teamwork and team morale was more pronounced among ward staff. Support was reciprocal between staff members, and some noted that this was the only support available at that time.

#### 3.4.2. Difficulties in Communication from Remote Working and Low Team Morale

A limited number of participants reported how, conversely, teamwork had been eroded during this time leading to a decline in team morale and cohesion. Some attributed this to limitations of remote communication in MDT, including lack of spontaneous interactions, tacit learning, social activities and face to face introductions of new members, but also imbalance in workload and engagement among team members. Further confounding factors included a lack of support from staff groups working remotely, depletion of personal energy in trying to keep team morale up and avoiding sharing personal struggles.

*I think communication, so that was really hard. Trying to communicate what we wanted, because obviously particularly during the first wave, everybody just disappeared. [] I think it was more to do with not being made to think that we were on our own, because that’s what it felt like.*

(Interview 6, senior sister)

#### 3.4.3. Need and Availability of Organizational Support

Organizational-level support was rarely mentioned in survey responses. Being informed by the organization and having an opportunity to feedback and input was seen as important for perceived cohesion and support. Interviewees’ perspectives varied regarding the expressed need for support. Some appeared unaware or disinterested in organizational support provisions, others discussed how they had or would welcome such support. Organizational support that had been utilised by interviewees was accessible within the renal clinics, rather at a general hospital-level.

*During the first wave of the pandemic, [] we were fortunate to have some group psychology sessions, and we were shown … [] the psychological pandemic model. And that was really helpful actually, and we were able to talk …*

(Interview 3, renal matron)

Coping behaviours varied and some turned to the team, family and friends, physical activity, outdoors and religion.

## 4. Discussion

The present study explored the impact of COVID-19 on kidney care delivery, quality and staff well-being, as seen by HCPs working in secondary kidney healthcare. The majority of participants referred to the rapid changes and adaptations, concerns about care quality, but also benefits from innovations; at staff-level, high work pressure, increased anxiety and mental exhaustion, but also the team as a well-being resource.

By the autumn/early winter 2020, when the majority of data collection took place, remote care had been fairly well established as the ‘norm’ and was expected to continue for the foreseeable future. Remote delivery acted as a mechanism in infection prevention, while allowing access to regular consultations for the clinically vulnerable kidney patients. However, the survey responses and interviews indicated challenges and shortcomings in care provision and care quality. Alongside the reduction of some services, remote patient monitoring proved challenging and was sometimes inadequate to capture disease progression and offer appropriate treatment in a timely manner, leading in some cases to acute deterioration. Findings are in line with existing research showing that telephone and video consultations are “less information rich” compared to face-to-face consultations [32], in turn impacting on information exchange, rapport and treatment planning which are all key functions of clinician-patient communication [33].

Technical difficulties and lack of IT hardware and infrastructure were also reported, a common finding in telehealth research [32,34]. Additionally, remote appointments may have exacerbated existing challenges in clinician-patient communication for certain patient groups, those for whom English isn’t a first language and those with disabilities such as hearing loss [35]. Evidence in favour of incorporating telemedicine in kidney care has been available prior to COVID-19 [25,26,27]. Present findings show that some applications of telemedicine were considered appropriate and advantageous, mainly consultations with stable and well-managed patients and as a method of triage. A two-sided account from HCPs emerged, i.e., support for integration of telemedicine in standard care vs. difficulties in the treatment of certain patient groups, particularly those whose conditions aren’t well managed and complex. This duality has been reported for other conditions [36,37,38] and in primary care [39].

Nevertheless, the findings highlighted efficiencies and innovations in kidney care. Some HCPs, for example, reported remote clinics were up and running by May 2020, while further adjustments in procedures were on-going. Telemedicine may have previously been perceived by HCPs and patients as a threat to traditional models of care. Its implementation required the deconstruction of traditional patient-clinician encounters in healthcare and redesigning of current models with boldness and vision [40], whilst real-life evidence on its effectiveness, particularly in kidney care, was limited [25,27]. Resistance to innovations for quality improvement is common in the complex healthcare systems [41]. Due to COVID-19, telemedicine and other innovations were rapidly implemented and trialled in this real world natural experiment offering a major opportunity to rapidly identify best practices and design new efficient models in renal care. Indeed, in our sample the majority of HCPs wanted to utilise telemedicine in the future through hybrid models using a combination of face-to-face and remote appointments where appropriate [29], while the majority of patients from the same settings and their significant others felt they could get the support they needed [28,29]. This confirms findings from a 4-year trial of referrals to virtual renal outpatient clinics that aimed to facilitate early access to specialist care in the UK which showed that up to one in four total referrals (with a trend for increase) were deemed eligible for virtual clinics, i.e., patients not at risk for disease progression [42].

On the other hand, the implications regarding staff well-being raised are a cause for concern. The majority of participants reported experiencing heightened stress and anxiety since the start of the pandemic, which was more pronounced among patient-facing staff. Numerous studies have demonstrated similar trends [8,10,11,12,43]. This is a typical response to a prolonged and highly stressful event, known as collective trauma [44]. What the findings demonstrate is there was a shift in mental burden expression as the pandemic unfolded, from an initial peak in stress [6,45], uncertainty [5] and overwhelming sense of helplessness for some HCPs [46], to weariness and mental exhaustion related to prolonged work pressure, moving to the second and third wave. This is particularly worrying given that HCPs in kidney services will most likely still have to deal with an increased workload from a backlog of care, deteriorating patients and COVID-19 patients with renal complications [47]. This can leave HCPs vulnerable to burnout, which is in turn linked to poorer care quality and patient safety [15,48]. The House of Commons report on workforce burnout in the NHS in May 2021 referred to an “extraordinarily dangerous risk” to its future functioning. The need for prioritization of HCPs well-being and provision of tangible and psychological HCP support has been highlighted since the beginning of the pandemic [6,16]. Increasing job resources and support to build individual HCPs resilience could buffer long-term impacts on both staff psychological wellbeing and care quality. This has been reflected in studies underpinned by the Job-Demands Resource model [9,49].

Besides formal support, peer support and teamwork were highlighted by many participants. Trusting relationships based on shared lived experiences and reciprocity are indeed important characteristics in peer mental-health support [50]. On the other hand, negative changes in team dynamics, the loss of opportunities for communication due to remote working and a lack of formal and informal face-to-face contact were also reported. Informal opportunities for knowledge sharing and watercooler communication are important in MDT learning [51]. When considering the continued use of telehealth in kidney services and how these are embedded in the wider health service system the impact on communication and tacit knowledge sharing, and team communication and clinical decision making needs to be taken into account.

**Figure 1:**
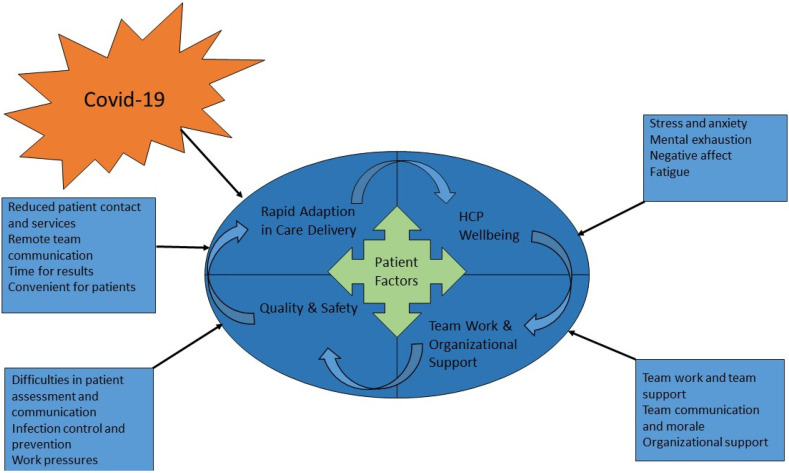
Socio-technical System Framework for Telemedicine in Kidney Care.

This study has highlighted there are a number of factors that need to be considered when implementing telemedicine in kidney care to ensure high-quality safe care is delivered to patients with kidney disease. The results of this study have been further interpreted into a theoretical socio-technical framework (Figure 1) of telemedicine in kidney services. This framework posits that the system needs to be understood in terms of both the social and technical aspects and these need to be brought together as interdependent parts [52]. Socio-technical frameworks have been used to understand telemedicine implementation in a number of clinical settings and consider the human, social, organizational and technical factors in the design and implementation of a system [53,54].

The technical factors in Figure 1 are related to the Rapid Adaptions in Care Delivery resulting in new technology implementation and remote appointments. This led to benefits and efficiencies for both HCPs through reduced patient contact and services, convenient appointments and rapid results, changes in remote team communication. The social factors include Team Work and Organisational Support these relate to how HCPs interact with each other through team support and team work, the differing need and availability of organisational support and the negative impact of remote working has had on team communication and morale.

Individual factors include the impacts on HCPs Wellbeing such as increased stress and anxiety and mental exhaustion, negative affect and fatigue. These technical, social and individual factors interact to influence patient Care Quality and Safety with the rapid technological adaptions leading to improved infection control and prevention but also some concerns for care quality due to increased work pressure and difficulties in patient assessments and communication. In this theoretical framework the patient is at the centre to ensure systems are designed to be patient centred and adapted appropriately to patient care needs.

This socio-technical framework can be applied in future research and service evaluations to improve both organizational and HCP resilience and care quality and safety. Future research should take account of these socio-technical factors and how these interact as interdependent parts of one system. Further applications could evaluate telemedicine implementation and ongoing adaptions and innovations in kidney services, particularly as telemedicine and/or hybrid models become the normalised mode of kidney care. Individual factors to consider include reducing HCP work pressure by addressing staff shortages, facilitating better work-life balance; and providing tailored HCP support both for individuals and teams. The technology-human interaction needs to be understood from both HCPs and patient perspectives to design remote consultations in ways that reduce patient fears and maximise the effectiveness of communication and clinical outcomes. Social factors of consideration are how HCPs teams can be involved in the development and implementation of innovations in renal care and how they can be supported effectively by the team and organization. Future adaptions of technical factors could consider how to triage patients, dependent on clinical needs and suitability for telemedicine. Technical factors need to consider what hardware and software infrastructure, such as online platforms, to facilitate optimum visual input from patients during clinical consultations is needed, the usability and acceptability of these and any potential data protection and security issues.

## 5. Strengths and Limitations

Strengths of the study are a good representation of renal HCPs with regard to occupation, age, work experience and geographic location (England); the qualitative inquiry of the survey allowing for in-depth exploration of the topic; the timing of data collection, i.e., past the initial phases of COVID-19 allowing for adaptations and reflection to take place; the rigour in data analysis and comprehensive theme presentation. A number of limitations also exist: the low participation of HCPs, i.e., only about 5% of invited HCPs participated in the survey, and subsequent limited sample size. In addition, many survey responses were brief. However, reports based on larger and/or different samples of HCPs reveal similarities in themes [1,11,12,38]. With regard to the interviews, it was not possible to confirm data saturation due to the limited number of available participants. Nevertheless, six interviews may suffice for themes to emerge [55] and interview data fit well with the survey coding structure. Overall, although HCPs’ experiences varied, the reported findings represent consistent patterns, discussed by the majority. Lastly, participants often did not make a distinction among different kidney conditions. Focused studies have given a condition-specific insight on COVID-19 impact, for example, emphasising the advantages of the increased home replacement therapy for dialysis patients [56] or the implications of interruption in living donation and kidney transplantation [57].

## 6. Conclusions

Moving forwards and taking into account the positive and negative impacts of COVID-19 found in the present study, balancing the efficiencies of remote appointments with care quality and safety are important considerations in NHS quality planning in kidney care. There is an unprecedented momentum for change and HCPs’ input is key in identifying the service model that best meets population needs [41]. Site-specific benefits and drawbacks should be gathered to allow for best practices to emerge. Present findings showed that telemedicine is feasible and suitable for several renal services and patient groups and its long-term retention can improve healthcare access and efficiency. Specification of conditions for its application is warranted. Renal HCPs have been dealing with high workload and anxiety and their support and engagement should also be components of quality planning. A socio-technical framework holds promise for identifying effective future adaptions and applications.

## Figures and Tables

**Table 1:** Participant characteristics.

	Survey Participants (*n* = 59)	Interview Participants (*n* = 8)
**Age**	43.7 (±11.4) years	47.1 (±7.3) years
**Gender** (Female)	31 (69.5%)	7 (87.5%)
**Ethnicity**		
White British	38 (64.4%)	5 (62.5%)
Asian	15 (25.4%)	2 (25%)
White Irish/Other	5 (8.4%)	1 (12.5%)
Black	1 (1.7%)	-
**Profession**		
Nurse	26 (44.1%)	5 (62.5%) *
Consultant/Registrar	16 (27.1%)	2 (25%)
Dietician	6 (10%)	1 (12.5%)
Research staff	3 (5.1%)	-
Other	8 (13.6%)	-

* senior sister/ward manager, inpatient matron, pre-dialysis sister, specialist nurse/home-dialysis, palliative care.

## Data Availability

The data presented in this study are available on request from the corresponding author. The data are not publicly available due to privacy restrictions.

## Supplementary Materials

The following are available online at https://www.mdpi.com/article/10.3390/ijerph19010188/s1, Supplement S1: Consolidated Criteria for Reporting Qualitative Research (COREQ) Statement, Supplement S2: Interview topic guide, Supplement S3: Table of themes, subthemes and original quotes.
