# Supplementary material for: Kidney Care during COVID-19 in the UK: Perspectives of Healthcare Professionals on Impacts on Care Quality and Staff Well-Being
Source: Int J Environ Res Public Health. 2021 Dec 24;19(1):188. doi: 10.3390/ijerph19010188 (PMC8750502; doi:10.3390/ijerph19010188)
Supplement: Supplementary file 1 [file ijerph-19-00188-s001.zip › ijerph-1418336-supplementary.pdf]

Supplement S1. Consolidated Criteria for Reporting Qualitative Research (COREQ) Statement

Section/Topic	Item No	Checklist item	Reported on page No
Domain 1: Research team and reflexivity			
Personal Characteristics			
Interviewer/facilitator	1	Which author/s conducted the interviews of focus groups Interviewer/facilitator	3
Credentials	2	What were the researcher credentials? E.g. PhD or MD	3/ Title page
Occupation	3	What was their occupation at the time of the study?	3/ Title page
Gender	4	Was the researcher male or female?	3
Experience and training	5	What experience or training did the researcher have?	3
Relationship with participants			
Relationship established	6	Was a relationship established prior to study commencement?	3
Participant knowledge of the interviewer	7	What did the participants know about the researcher? E.g. personal goals reasons for doing the research	3
Interviewer characteristics	8	What characteristics were reported about the interviewer/facilitator? e.g. Bias, assumptions, reasons and interests in the research topic	3
Domain 2: Study design			
Theoretical framework			
Methodological orientation and Theory	9	What methodological orientation was stated to underpin the study? E.g. grounded theory, discourse analysis, ethnography, phenomenology, content analysis	3
Participant selection			
Sampling	10	How were participants selected? E.g. purposive, convenience, consecutive, snowball	3
Method of approach	11	How were participants approached? E.g. face to face, telephone, email	3
Sample size	12	How many participants were in the study?	3-4
Non-participation	13	How many people refused to participate or dropped out? Reasons?	3
Setting of data collection	14	Where was the data collected? E.g. home, workplace, clinic	3
Presence of non-participants	15	Was anyone present besides the participants and the researchers?	3
Description of sample	16	What are the important characteristics of the sample? E.g. demographic data, date	Table 1
Data collection			
Interview guide	17	Were questions, prompts, guides provided by the authors? Was it pilot tested?	3, Suppl. 2
Repeat interviews	18	Were repeat interviews carried out? If yes how many?	3
Audio/visual recording	19	Did the research use audio or visual recording to collect the data?	3

Field notes	20	Were field notes made during and/or after the interview or focus group?	3
Duration	21	What was the duration of the interviews or focus group?	4
Data saturation	22	Was data saturation discussed?	11
Transcripts returned	23	Were transcripts returned to participants for comment and/or correction?	n/a
Domain 3: Analysis & Findings			
Data Analysis			
Number of data coders	24	How many data coders coded the data?	3
Description of the coding tree	25	Did authors provide a description of the coding tree?	Suppl. 3
Derivation of themes	26	Were themes identified in advance or derived from the data?	3
Software	27	What software if applicable was used to manage the data?	3
Participant checking	28	Did participants provide feedback on the findings?	n/a
Reporting			
Quotations presented	29	Were participant quotations presented to illustrate the themes/findings? Was each quotation identified? E.g. participant number	4-8, Suppl. 3
Data and findings consistent	30	Was there consistency between the data presented and the findings?	4-8, Suppl. 3
Clarity of major themes	31	Were major themes clearly presented in the findings?	4-8, Suppl. 3
Clarity of minor themes	32	Is there a description of diverse cases or discussion of minor themes?	4-8

Supplement S2. Interview topic guide

Introduction

1. Could you tell me a bit about your role? Has this changed during the pandemic in any ways?

Impact on kidney care

2. Could you tell me about your experience of working as [ ] during a pandemic? How has it been for you?
3. What has been most challenging for you since last March? As a professional and in general. Has any part of your experience been particularly painful or traumatic?
4. How was your experience with remote care?
5. Thinking of the changes in NHS and your clinical practice since last March, what did you find worked well? What could be improved?
6. Is there any kind of support that you would have welcomed, but wasn't available?
   Prompts: any training, for example with remote care? Support for wellbeing?
7. At a personal level, how have you coped during the pandemic? How have you looked after yourself, your wellbeing?
8. How do you find it has been for your patients? Has it been more difficult for any specific patient groups?
   Prompts: How have patients responded to remote healthcare? Any resources you think would be helpful for the patients? What is the role of family members in remote healthcare?

Moving forward

9. Moving forward, how do you see the future in kidney care? How do you think kidney care will be delivered in the future? What would you keep of the changes, what should go back to how it was?
   Prompts: What you think the role of remote care will be? What are the short- and long-term plans by the Trust and NHS? What support would you find useful?

Patient self-management

10. What can kidney patients do to take care of themselves under the circumstances? How important? Is this different from before the pandemic? Is this something you discuss in your appointments?
11. What do you consider a potentially effective way of promoting and supporting self-management at present? Have you referred patients to any (online) resources for self-management support?
    Prompts: if haven't referred patients- how come? Is this something that would fit your practice as it is? What would make you or convince you to? Any specific outcomes you would like to see?

Closing

12. Is there anything you would like to add, anything I've missed?

Supplement S3. Table of themes, subthemes and original quotes

**Themes**	**Subthemes, second order**	**Subthemes, first order**	**Quotes**
**1. Rapid changes and adaptations in care delivery**		Reduced patient contact and services<br><br>(38 codes*)	*Yes, far more phone consultations, reduced visiting and reduced face to face contact with patients where possible* (nurse, 46y*)*<br>*Pre-treatment information given initially by phone. Signposting to web sites Posting out written information OPD appointments via telephone* (nurse, 59y*)*<br>*We do however continue to see patients for treatment purposes or if we feel they have had a deterioration in their clinical health and wellbeing. (nurse, 31y)*<br>*During the summer when case numbers were low, the threshold for arranging face to face appointments dropped and higher numbers were attending clinic. Now we are into the second wave, we have had to cut back on that. (doctor, 38y)*<br>*For the first 7 weeks following lockdown no visits were made to the haemodialysis unit.* (dietician, 59y)
		Remote team communication<br><br>(10 codes)	*More virtual than face to face interaction*. (doctor, 38y)<br>*Face-to-face meeting with members of study team has been stopped. We communicate using zoom, emails and phone, etc. We are still able to have proper communication with the help of different technology* (nurse, 46y)
		Infection control and prevention<br><br>(6 codes)	*Increased awareness with social distancing, PPE requirements, self-awareness with potential symptoms* (nurse, 47y)<br>*And even now we have a thermometer and a sats machine, a portable one, so every time we go inside we need to make sure that everything is safe. So yeah, that changed. (I1, specialist nurse)*
**2. Quality of care**	2.1 Drawbacks and concerns for care quality	Difficulties in patient assessment and communication	*However, for some patients telephone clinics mean problems are not being sorted, not getting monitoring to know if intervention is required* (doctor, 31y)

		(57 codes)	*Tele-clinics less safe for AKC patients. Remote supervision of HD patients time wasteful and less safe* (doctor, 73y)<br>*Patients not admitting to symptoms on the phone and becoming unwell leading to acute episodes, visits to renal assessment unit and admission. Patients starting renal replacement therapy sooner than expected as they have not been monitored as effectively* (nurse, 59y)<br>*While I still feel I offered an effective service, it is not the same when you cannot see the patient and pick up on non-verbal communication and just see how they are looking* (dietician, 50y)<br>*Subsequently we were unable to review them [i.e., patients in wards] and check they were on an appropriate treatment plan*. (nurse, 31y)<br>*The majority of dialysis patients were dialysing for fewer hours, which for some meant increasing dietary or fluid restrictions further, potentially impacting on quality of life* (dietician, 58y)<br>*They [dialysis patients] tend to be complex, some have hearing difficulties, and others are non-English speakers. I feel that I cannot give them the level of care that I would be able to if I were visiting the unit / seeing them face to face* (doctor, 38y*)*<br>*Building rapport is key to our assessment being patient friendly. The physical barrier of the mask and visors do not help us reassure our patients of confidentiality as they divulge very personal information on a ward* (occupational therapist, 51y)
		Increased work pressure<br><br>(21 codes)	*Increased work load, more paper works and chasing requests; like blood results and referrals (nurse, 49y)*<br>*Unable to deliver group sessions to patients therefore we needed to review the PTL and pull patients into 1:1 sessions. Additional home visits were required to assess patients. This was more time consuming on our team and also we had limited resources to be able to carry this out (nurse, 31y)*<br>*Everyone is 'busier' - it is certainly a perception and it has factual basis eg staffing challenges due to sickness/isolation. Serious nursing shortage; pressured demand for 'bank' working (doctor 73y)*

			Huge pressure due to no coordination of team efforts (doctor, 31y)<br>*We are now called into work out of hours much more frequently and for longer periods of time. (biomedical scientist, 27y)*
	2.2 Efficiencies and benefits	Efficiencies in clinical practice<br><br>(28 codes)	*Managing patients by blood tests and telephone calls has been easier for some of them. Perhaps highlighted we can see some patients less frequently so long as they have regular blood tests. Less need for patient transport*. (nurse, 59y)<br>*Also helps more doctors to be available on the main site while they can perform dual roles while doing virtual clinics* (doctor, 38y)<br>*We have learned much more about working from home and using remote technology. I certainly think we will be reducing travel to meetings in the future* (doctor, 60y)<br>*I mean what the pandemic has shown is that we can work with primary care very quickly and very effectively* (I8, consultant)
		Crisis enabling innovation<br><br>(8 codes)	*Yes, we found out that not every consultation needs to be face to face* (doctor, 33y)<br>*we haven't had a lot of choice this year, and I think it's forced us to think a little differently about our roles, and moving forward we probably won't go all the way back to how we used to work* (I7, dietician)<br>*And the guy that* [created a new service]*, he's been wishing to do it for eight years, he's been saying this'll be amazing for patients*, (I5 specialty doctor)
		Covid-19 safety<br><br>(2 codes)	*The changes have benefited both staff and patients in the fact of reducing the risk factor of the virus* (nurse, 55y)<br>*Patients were shielding so were reluctant to come to appointments and enjoyed not having to travel to appointment* (dietician, 50y)<br>*Well, you have to be careful what you do, what you clean, how do you clean. There's so many things that you're looking now, that you never looked before. I don't know, it is going to be very difficult to come back as before*. (I1, specialist nurse)
3. Staff well-being		Increased stress and anxiety	*Higher levels of general stress amongst the clinical team.* (doctor, 39y)

		(30 codes)	*Honestly, we became paranoid and disinfect everything. Scared for my family what I will bring home I am not scared really but more threatened if I give it to my family.* (nurse, 49y)<br>*I am anxious about patients being unwell at home and not seeing them regularly.* (nurse, 59y)<br>*There is anxiety relating to uncertainty and a demoralization as so many planned activities are cancelled and contact with friends and family is reduced* (doctor, 60y)<br>*I mean dealing with Covid patients is quite distressing, because the therapies are quite limited, and obviously… And not that death is ever pleasant, but some of the death that's going on is quite… These people are very lonely, they're in hospital, so that's quite difficult to see that. And the therapeutic options for many people are quite limited, so that's been quite challenging kind of professionally.* (I8, consultant)
		Mental exhaustion, negative affect and fatigue<br><br>(28 codes)	*Initially, the team bonded and worked really well together. As the months have rolled on, people's resilience and mental health have been tested* (nurse, 37y)<br>*I feel exhausted after shifts and have worked late on regular basis to try and keep up. I have booked more random days off work just to recuperate. I take pain relief every day without fail.[] General unhappiness is the new normal sadly*. (occupational therapist, 51y)<br>*…it's the physically feeling tired, but mentally drained, because the shift is just full-on, and working over your hours. Which we did get paid overtime for until the end of June, and then they stopped because of our grade.* (I3, matron)<br>*it's getting too much sometimes, there's too much information. But it's there, it's every day, so it comes as a normal thing now. Don't know, cry sometimes, but nothing… Yeah I mean I think I'm fine.* (I1, specialist nurse)
4. Social and organisational support		Team support and teamwork<br><br>(25 codes)	*Team work very hard but remain upbeat and supportive of each other*. (nurse, 59)<br>*Team morale and teamwork improved - felt there was a sense of `this is a crisis so we have to get on with it`* (ward manager, 46y)<br>*Everyone stepped up when required and was flexible, but we have a good team anyway.* (doctor, 55y)

			I don't suppose we've had support from anybody else. I suppose… I don't think any… I think we've all been in the same boat. You know, everywhere is the same in the hospital, we're all kind of like short-staffed, because people are shielding or people are having to isolate. Everybody's just had to pull together. (I2, pre-dialysis sister)<br>*But looking back, I think that they did the best teamwork that they've ever done in that period of time. You know, and seemed to keep themselves rallied.* (I6, senior sister)
		Difficulties in communication and low team morale<br><br>(24 codes)	*Team initially worked together but then as it became apparent that some were working much harder than others the team has become more disjointed* (doctor, 39)<br>*Time out with 'stress' more common among the MDT. The opportunities for humour, exhortation through leadership are fewer and more compressed. Corridor chats were always good for morale.* (doctor, 73)<br>*Most of the time this [remote communication] is adequate, though as the months have passed I have gradually felt more "cut off" from the team* (doctor, 38)<br>*I think communication, so that was really hard. Trying to communicate what we wanted, because obviously particularly during the first wave, everybody just disappeared. [] I think it was more to do with not being made to think that we were on our own, because that's what it felt like*. (I6, senior sister)<br>*Via email, letter and handover. Not much changed* (nurse, 32)
		Need and availability of organisational support<br><br>(36 codes- interviews)	*So the command and control structure I think worked really really well at the beginning, until we were getting… Started to get exhausted with it. It was quite reassuring that you've got the whole escalation through the command and control structure, and you felt that your voice was being heard, and you definitely felt support coming back down.* (I3, renal matron)<br>*I think for the staff, the hospital has some kind of, yeah, well-being team. If you are struggling or you have any problems you always can contact them. But I don't know, I never use it.* (I1, specialist nurse)<br>*I personally think somebody actually saying to you are you alright? []I think no-one really checked if we were OK in what was going on. I mean some people probably didn't need it,*

			and others needed it more, but I think sometimes if somebody'd said are you OK? (I2, pre-dialysis sister)<br>*During the first wave of the pandemic, myself and two [] sisters, we were fortunate to have some group psychology sessions, and we were shown… All done on Teams meetings, we were… Microsoft Teams, we were shown the psychological pandemic model. And that was really helpful actually, and we were able to talk...* (I3, renal matron)<br>*I think I have been really kind of a bit stressed and overwhelmed at times, but like only within what you would expect for the situation, and I didn't feel particularly that I needed support. [] I think there was lots and lots of stuff in the Trust available, you know, quiet rooms and all that kind of thing to people that were working on the wards and needed a bit of time, and I think we had lots of access to sort of psychological therapies and stuff that we can get through work (I5, specialty doctor)*<br>*No, the only support I think would have been beneficial was I think I found… As a team leader I found out about a lot of things that were happening nationally after the event. [] But other than that, I think the support for the change of the service or the professional way of doing things, I think, you know, it's been great* (I7, dietician)<br>*…there's a duty of care for your employer to look after your employees. So I don't think any employee, or patient actually, should be catching Covid in hospital []*<br>*So there wasn't anything extra I… I don't feel training was an issue, it was more around, as I say, resource management and personal protection that I thought were the big issues for me.* (I8, consultant)

* Number of codes at the second phase of the coding process, following comparison and merging of initial codes
